# Supplementary material for: Effects of sake lees intake on fecal uremic toxins, plasma D-alanine, constipation, and gut microbiome in healthy adults: A single-arm clinical trial
Source: PLoS One. 2025 Jun 3;20(6):e0325482. doi: 10.1371/journal.pone.0325482 (PMC12133169; doi:10.1371/journal.pone.0325482)
Supplement: S1 Table — Data are presented as mean values (SD). Nutritional intake exludes nutrients derived from sake lees. CSS, Constipation Scoring System; PAC-QOL, Patient Assessment of Constipation Quality of Life. (DOCX) [file pone.0325482.s005.docx]

|  | Overall (n = 8) | | | | Sake lees 25 g (n = 4) | | | | Sake lees 50 g (n = 4) | | | | |
| --- | --- | --- | --- | --- | --- | --- | --- | --- | --- | --- | --- | --- | --- |
|  | Baseline | Week 2 | Week 4 | Week 6 | Baseline | Week 2 | Week 4 | Week 6 | Baseline | Week 2 | Week 4 | Week 6 |  |
| Measured values |  |  |  |  |  |  |  |  |  |  |  |  |  |
| Fecal indole, µg/g | 45 (26) | 63 (67) | 53 (38) | 43 (40) | 39 (33) | 43 (23) | 26 (15) | 27 (14) | 52 (20) | 85 (94) | 80 (34) | 58 (55) |  |
| Fecal p-cresol, µg/g | 130 (37) | 151 (109) | 110 (59) | 107 (50) | 118 (39) | 110 (90) | 83 (73) | 83 (42) | 141 (35) | 191 (124) | 137 (27) | 132 (49) |  |
| Plasma D-alanine, nmol/mL | 0.89 (0.36) | 1.16 (0.47) | 1.06 (0.44) | 1.17 (0.49) | 0.72 (0.19) | 0.93 (0.24) | 0.77 (0.24) | 0.85 (0.36) | 1.10 (0.43) | 1.40 (0.55) | 1.36 (0.40) | 1.49 (0.41) |  |
| Plasma D-serine, nmol/mL | 2.22 (0.79) | 2.29 (0.82) | 2.02 (0.64) | 2.02 (0.76) | 1.84 (0.25) | 1.97 (0.20) | 1.79 (0.06) | 1.97 (1.70) | 2.60 (1.02) | 2.61 (1.22) | 2.26 (0.91) | 2.27 (1.07) |  |
| CSS score, point | 9.2 (3.0) | 6.8 (2.3) | 7.3 (2.7) | 7.4 (4.5) | 8.8 (1.3) | 5.5 (2.4) | 5.7 (2.4) | 5.8 (2.9) | 9.8 (4.3) | 8.0 (1.4) | 8.8 (2.2) | 9.0 (5.7) |  |
| PAC-QOL score, point | 43 (15) | 25 (15) | 27 (14) | 25 (18) | 40 (15) | 18 (9) | 17 (8) | 13 (8) | 46 (18) | 32 (19) | 38 (10) | 38 (17) |  |
| Nutritional intake |  |  |  |  |  |  |  |  |  |  |  |  |  |
| Energy, kcal/day | 1784 (332) | - | - | 1866 (355) | 1804 (282) | - | - | 1939 (319) | 1764 (422) | - | - | 1793 (423) |  |
| Protein, g/day | 64 (17) | - | - | 63 (17) | 56 (11) | - | - | 53 (11) | 71 (20) | - | - | 74 (16) |  |
| Salt, g/day | 9.2 (3.1) | - | - | 9.0 (3.2) | 7.6 (0.6) | - | - | 7.4 (1.1) | 10.9 (3.8) | - | - | 10.4 (4.1) |  |
| Dietary fiber, g/day | 12.5 (3.7) | - | - | 13.0 (3.5) | 11.1 (1.3) | - | - | 11.6 (1.4) | 13.9 (5.0) | - | - | 14.3 (4.3) |  |
| Change from baseline (%) |  |  |  |  |  |  |  |  |  |  |  |  |  |
| Fecal indole | 0 (reference) | +42 (94) | +52 (146) | −6 (51) | 0 (reference) | +39 (81) | −1 (82) | −14 (40) | 0 (reference) | +46 (120) | +106 (189) | +2 (65) |  |
| Fecal p-cresol | 0 (reference) | +7 (73) | −20 (39) | -20 (33) | 0 (reference) | −15 (74) | -40 (52) | -32 (20) | 0 (reference) | +28 (74) | −22 (7) | −7 (22) |  |
| Plasma D-alanine | 0 (reference) | +39 (29) | +24 (36) | +38 (50) | 0 (reference) | +34 (43) | +13 (49) | +26 (64) | 0 (reference) | +34 (14) | +34 (20) | +50 (40) |  |
| Plasma D-serine | 0 (reference) | +4 (14) | −7 (10) | −8 (13) | 0 (reference) | +9 (18) | −2 (10) | −3 (12) | 0 (reference) | 0 (9) | −13 (8) | −14 (13) |  |
