# Supplementary material for: Effects of sake lees intake on fecal uremic toxins, plasma D-alanine, constipation, and gut microbiome in healthy adults: A single-arm clinical trial
Source: PLoS One. 2025 Jun 3;20(6):e0325482. doi: 10.1371/journal.pone.0325482 (PMC12133169; doi:10.1371/journal.pone.0325482)
Supplement: S1 Protocol — (DOCX) [file pone.0325482.s006.docx]

Created on October 8, 2021 Ver. 1.0

**Research plan**

**1. Title**

Effects of sake lees intake on fecal uremic toxins, plasma D-alanine, constipation, and gut microbiome in healthy adults: A single-arm clinical trial

**2. Outline, Purpose and Significance of the Study**

**Summary**

The efficacy of sakekasu intake on the intestinal environment in patients with chronic constipation will be evaluated in a single-arm (2-dose) intervention study. The primary endpoint will be uremic substances in stools, and secondary endpoints will be bacterial flora and short-chain fatty acids in stools.

**Objective**

The study described in this research protocol (hereafter referred to as "this study") is a pilot study aimed at evaluating changes in metabolites and bacterial flora in stools by amount and duration of sake-kasu intake in patients with chronic constipation. Based on the results obtained in this study, the amount and duration of sake lees intake in a small randomized controlled trial (RCT) in patients with chronic kidney disease (CKD) will be established.

**Background**

Patients with CKD are known to have dysbiosis (DysB), a condition in which the intestinal microbiota is disrupted^1)^ . DysB occurs in CKD patients due to elevated urea and uric acid in the blood that migrate into the intestinal tract, medication, and dietary fiber deficiency due to dietary restriction. These decrease beneficial strains that produce short-chain fatty acids, which are a source of energy for the intestinal epithelium and involved in intestinal immune regulation^2)^ , and conversely increase harmful strains that produce uremic substances such as indoles and cresols^3)^ . In other words, the metabolites produced by DysB are altered, and a vicious cycle may be formed in which CKD develops^4)^ . For example, deficiency of short-chain fatty acids causes weakening of the intestinal epithelial barrier, and pathogens and uremic substances that should normally remain in the intestinal tract may pass through the intestinal epithelium and enter the bloodstream, leading to renal damage via chronic inflammation and other factors.^5)^

This makes regulation of the gut microbiota and metabolites in CKD patients important in the prevention of CKD progression. Therefore, factors involved in the regulation of gut microbiota and metabolites need to be clarified.

Diet is a factor involved in the gut microbiota and metabolites, and the common treatment of DysB is a probiotic (intake of beneficial bacteria, ProB) and prebiotic (intake of dietary fiber, PreB) diet, with ProB and PreB being tested in patients with CKD. ProB and PreB are being tested in CKD patients. For example, ProB/PreB in CKD patients has been reported to decrease blood levels of uremic substances (p-cresyl sulfate).^6)^ Thus, ProB/PreB suitable for CKD patients may be a useful DysB treatment.

Sake lees contains beneficial bacteria and dietary fiber, and has both ProB and PreB functions suitable for CKD patients. In addition, it contains low amounts of phosphorus and potassium, which are restricted in CKD patients, and low amounts of salt. As for its effects, studies on people with constipation have reported that consumption of 190 g of amazake (equivalent to 25 g of sakekasu) for 4 weeks improves the intestinal microflora^7)^ and 50 g of sakekasu for 3 weeks improves constipation^8)^ .

D-serine and D-alanine, a type of D-amino acid, have been confirmed to have renoprotective effects by research members^9)^ . Fermented foods such as sake-kasu contain D-amino acids, and direct intake of D-amino acids from sake-kasu may provide renoprotective effects. However, there are no studies that have examined changes in D-serine and D-alanine in stool and blood due to intake of sakekasu. In addition, there are no studies on changes in stool flora related to metabolites such as uremic substances and short-chain fatty acids among the previously reported studies on sake-kasu intake and stool flora, and there is a lack of data on evaluation indices to show the usefulness of sake-kasu intake.

Therefore, the purpose of this study is to evaluate changes in metabolites and bacterial flora in stools by amount and duration of sake-kasu intake in patients with chronic constipation without CKD. Based on the results obtained in this study, we will establish the amount and duration of sake lees intake in a small RCT of patients with CKD.

**3. Scientific rationale and basis for the research**

**Study summary and primary endpoints**

The subjects of this study will be patients with chronic constipation and the intervention will be consumption of 25 g/day or 50 g/day of sake-kasu. The control will be pre-intervention, and the primary endpoint will be the percentage change in the concentration of uremic substances (indole and cresol) in the stool after 2, 4, and 6 weeks. The study design will be a single-arm (2-dose) pre/post comparison.

**Validity of the study design**

The subjects of this study shall be patients with chronic constipation without CKD. So far, there are no studies using sakekasu for CKD patients as far as we could find, and there are a few studies on patients with chronic constipation. There have been reports of an increase in certain species of bacteria that are considered beneficial bacteria after consumption of sake lees,^7)^ and the effect of improving constipation,^8)^ . Since changes in the intestinal microbiota and uremic substances can be inferred from sake lees consumption, and these changes can be expected to be observed even in those without impaired renal function, patients with chronic constipation without CKD should be included in this study.

The intervention will be intake of 25 g/day or 50 g/day of sakekasu for 6 weeks. In previous reports, studies on subjects with constipation have reported improved intestinal microbiota after 4 weeks of intake of 190 g of amazake (equivalent to 25 g of sakekasu)^7)^ and the effect of 3 weeks of intake of 50 g of sakekasu on constipation^8)^ . In addition, a study of CKD patients reported a decrease in blood uremic substance levels after 6 weeks of synbiotic supplementation^10)^ . Based on these results, the intervention in this study will be the intake of 25 g/day or 50 g/day of sake-kasu for a total of 6 weeks, and based on the results obtained, the amount and duration of sake-kasu intake in a small RCT of CKD patients will be established.

The method of outcome evaluation will be a before-and-after comparative study with no comparators. This study is conducted to obtain the rationale for setting the amount and duration of sake lees intake in a small RCT of CKD patients. Since changes in uremic substances in stools can be confirmed by pre- and post-intervention comparisons, this is a single group (2 doses) pre- and post-comparison study without a comparison group. Evaluation will be conducted 4 times at intervention, 2 weeks, 4 weeks, and 6 weeks after the intervention, and the amount and duration of intake of sake lees in a small RCT will be set.

**Alignment with study objectives and primary endpoints**

The purpose of this study is to confirm the change of uremic substances in stool, which is the process in observing the change of uremic substances in blood by intake of sake lees for CKD patients, which is the ultimate goal of this study. There are several studies on changes in blood uremic substances using ProB and PreB supplements. For example, a study of ProB/PreB supplementation and its effect on blood uremic substances (p-cresyl sulfate) in patients with CKD stages 3 to 4 reported a significant decrease in p-cresyl sulfate after 2 and 4 weeks.^6)^ A similar study in non-dialysis patients with CKD stages 4 to 5 also showed a decrease in p-cresyl sulfate after 6 weeks.^10)^ These results favor a mechanism by which ProB/PreB altered the gut microbiota and gut metabolites, thereby reducing uremic substances in the blood. To elucidate this mechanism, it is necessary to confirm the uremic substances in stools after intake of sake lees, and the purpose of this study can be achieved by evaluating the primary endpoints. In healthy subjects with normal renal function, blood uremic substances are not evaluated in this study because no change in blood uremic substances is expected to occur before and after intake of sake lees.

**4. Research Subjects and Selection Policy**

**(1) Eligibility criteria**

1. Healthy subjects who meet the diagnostic criteria^11)^ for constipation from the date of approval through January 2022

1. diagnostic criteria for constipation

Satisfy at least two of the following six criteria

1. Strong straining is required for more than one-fourth of bowel movements.
2. More than one-fourth of defecations are fecal or hard (type 1 or 2 on the Bristol stool shape scale).
3. More than one-fourth of the time, the patient feels a sensation of residual stools.
4. More than one-quarter of defecations are accompanied by a sensation of rectal obstruction or dyspareunia.
5. Manual assistance is required for more than one-fourth of bowel movements (e.g., stool removal, perineal compression, etc.).
6. Spontaneous defecation frequency is less than 3 times per week.

2. diagnostic criteria for "chronic

Symptoms present for at least 6 months and meeting the above criteria for the last 3 months

1. Persons who give written consent to participate in this study
2. Persons who are 20 years of age or older at the time of obtaining consent

**(2) Exclusion criteria**

1. Persons with alcohol intolerance
2. Have a history of antimicrobial use within the past 4 weeks from the time of study entry
3. Those taking drugs with side effects of diarrhea or constipation.
4. Other persons who are judged by a physician to be unsuitable for the subject.

**5. Target number and study period**

**(1) Target number**

Overall target number of cases eight

**(2) Research period**

Research period: Date of approval - March 31, 2023

Registration period: Date of approval - January 31, 2022

Observation period (follow-up period): 3 months after the end of enrollment

Analysis period: Date of approval - March 31, 2023

**6. Research methods**

**(1) Study design**

Single arm (2 doses) before/after study (single center)

**(2) Research Methods**

**Recruitment of research participants**

Kanazawa University staff will be notified via bulletin boards and mailing lists within 　Kanazawa University, and participants, including family members of staff, will be invited (excluding those involved in this study and their families).

**Allocation of research subjects**

The principal investigator will assign the amount and duration of sake-kasu intake to the research subjects. The amount and duration of intake of sake lees will be assigned in numerical order for each subject enrolled in the study.

**Research Procedures**

1. Provide nutritional guidance prior to intervention using our own sake-kasu recipe (Attachment 1).
2. Sake lees 25 g/day or sake lees 50 g/day for 6 weeks
3. Sakekasu (sake lees) from "Kakuma-no-sato", a refined sake sold by Kanazawa University, will be used. The same lot of sakekasu will be provided to the participants free of charge in order to ensure the same conditions for the intervention.
4. Store sake lees in a refrigerator at about 5℃.
5. Participants can choose how to cook sake-kasu from a collection of sake-kasu recipes
6. Outcome items will be assessed at intervention, 2, 4, and 6 weeks
7. The intake of sake lees and defecation should be recorded daily on the record sheet.
8. The timing and frequency of sakekasu intake is not set. For example, 25 g of sakekasu can be consumed in one meal or divided into three meals.
9. During the introduction period (2 weeks) and the intervention period (6 weeks), the consumption of lactic acid bacteria preparations, foods for specified health uses and foods with functional claims that improve the intestinal environment is prohibited. (Fermented foods other than sake lees, such as miso and yogurt (general foods), may be consumed.
10. The same dinner (a frozen home-delivered lunch with controlled nutritional value) on the day before the evaluation
11. Breakfast on the day of evaluation after 2, 4, and 6 weeks will be the same meal containing sakekasu

**Sample Acquisition**

Samples will be obtained by the following methods a schedule of actions will be prepared separately for the participants, describing how and where the stool samples will be submitted, and will be explained to them at the time of obtaining their consent.

1. Stool samples are taken at intervention, 2, 4, and 6 weeks after sake lees consumption to obtain concentrations of uremic substances in stool, short-chain fatty acids, and bacterial flora in stool.
2. Urine samples at intervention and after 6 weeks to obtain urinary pH.
3. Stool and urine samples will be collected at the participant's home using the prescribed kit.
4. At intervention, 2, 4, and 6 weeks after sake lees consumption, blood samples are taken to obtain blood D-amino acid levels.
5. The examinee records the status of sake lees intake daily using an independently prepared record sheet (Appendix 2).
6. Daily recording of defecation frequency and stool characteristics by the examinee using the Bristol Scale^12)^ (Appendix 3).
7. Validated dietary assessment instruments will be administered 2 weeks prior to the intervention and at the end of the intervention (6 weeks later) to assess food intake frequency and nutrient intake.

*4 involves a minor invasion when collecting blood samples. 1, 2, 3, 5, and 6 do not involve physical invasion since they are obtained through stool and urine samples and questionnaires.

**Location of the study**

The examination, blood sampling, and nutritional guidance will be conducted in the Laboratory of Nephrology, Kanazawa University Graduate School of Nephrology. Body composition measurement will be conducted in the Nutritional Counseling Room of Kanazawa University Hospital.

**Data Analysis Method**

The primary endpoint, the concentration of uremic substances in the stool, will be analyzed before and after the intervention using analysis of variance and Dunnett test, two-tailed, at a 5% level of significance.

**Disclosure of Research Results**

At the time of obtaining consent, participants will be asked to confirm their willingness to disclose the results of the research, and if they wish to do so, the results of stool, urine, and blood tests will be disclosed. The method of disclosure will be in writing to the research participant. However, if the request for disclosure is cancelled during the course of the research, no disclosure will be made.

**7. Observation, examination and report items**

Observation and inspection items shall be as follows

**Background of the examinee**

Age (year of birth), gender, height, weight, BMI

**Stool (fecal) examination**

Uremic substances (indole, p-cresol), flora, short-chain fatty acids (acetic acid, lactic acid, propionic acid, butyric acid), pH

**Urinalysis**

　pH (measure of acidity)

**Blood test**

D-Serine, D-Alanine

**Frequency of defecation, stool characteristics**

Bristol Scale, Constipation and Quality of Life Score (PAC-QOL)^13)^  (Exhibit 4)

**Food Intake Frequency and Nutrient Intake Assessment**

Food Frequency of Intake Survey (FFQg)^14)^ (Exhibit 5), Compliance with Liquorice Consumption

**Body composition measurement**

Skeletal muscle mass, trunk muscle mass, limb muscle mass, body fat percentage, body fat mass, body cell mass, extracellular water ratio

**Schedule of Observations and Inspections**

The range of the inspection period shall be no more than 5 days before or after the reference date.

| **Reference Date** | **Before intervention  (-2 weeks)** | **time of intervention** | **2 weeks later** | **4 weeks later** | **After 6 weeks  （at the end of intervention)** |
| --- | --- | --- | --- | --- | --- |
| Visit | **1** | **2** | **3** | **4** | **5** |
| Doctor's Examination | **●** |  |  |  |  |
| Obtaining Consent | **●** |  |  |  |  |
| nutritional guidance | **●** | **●** | **●** | **●** | **●** |
| Food Intake Frequency Survey | **●** |  |  |  | **●** |
| Constipation and Quality of Life Score | **●** | **●** | **●** | **●** | **●** |
| Body composition measurement |  | **●** | **●** | **●** | **●** |
| Stool (fecal) examination |  | **●** | **●** | **●** | **●** |
| Urinalysis |  | **●** |  |  | **●** |
| Blood test |  | **●** | **●** | **●** | **●** |
| Sake Lees intake status |  |  | **every day** | | |
| Frequency of defecation, stool characteristics |  |  | **every day** | | |

**8. evaluation and reporting of adverse events**

**(1) Definition of adverse events and reporting methods**

☒ N/A

☐ Yes

This study involves minor invasive procedures as blood samples will be drawn before and after the intervention. The condition of the study subjects will be thoroughly observed during the blood draw, and the blood draw will be stopped if there is any discomfort.

Sake lees is a food that is consumed on a daily basis, and adverse events are not expected to occur due to its consumption. However, in the event of an undesirable change in physical condition, the researcher should consult with the physician in charge of the research through the contact person and take appropriate measures. If the physician determines that it is difficult to continue the research, the consumption of sake lees will be discontinued and the matter will be reported to the department head.

**(2) The burdens and anticipated risks and benefits to the research subjects, a comprehensive assessment of these burdens and risks, and measures to minimize such burdens and risks**

**(1) Projected profit**

There will be no direct benefit to individual research subjects from participating in this study. Research results may contribute to the advancement of medical care. As a reward, a QUO card (up to 20,000 yen) will be given in accordance with the University's regulations at the end of the research.

Sake lees will be provided free of charge to the research subjects because it is necessary to use the same product and the same lot of sake lees in order to match the intervention conditions.

**(2) Anticipated risks and disadvantages**

This study involves minor invasive procedures as blood samples will be drawn before and after the intervention. The condition of the study subjects will be thoroughly observed during the blood draw, and the blood draw will be stopped if there is any discomfort.

Since sake lees contain trace amounts of alcohol, driving a car after consumption of sake lees with insufficient alcohol volatilization may constitute driving under the influence of alcohol. The use of sakekasu with sufficiently volatilized alcohol by adhering to the cooking method according to the protocol should be emphasized in nutritional guidance during the intervention.

**9. Evaluation items**

**(1) Primary endpoint**

Percent change in concentrations of uremic substances (indoles and cresols) in stool

**(2) Secondary endpoints**

- Percentage change in the percentage of major bacterial species in stool
- Concentration of short-chain fatty acids (acetic acid, lactic acid, propionic acid, butyric acid) in stool
- Stool pH, stool characteristics, frequency of defecation
- D-amino acid concentration in blood
- Defecation frequency and characteristics, constipation and quality of life score (PAC-QOL)
- Frequency of food intake, amount of nutrients consumed, and percentage of compliance with sake lees

**10. Statistical matters**

**Number of cases design**

Eight cases were set based on feasibility.

**11. Filling out and reporting case reports**

The case report form should include the basic attributes of the research participant, various laboratory data during the intervention period, findings of the physician in charge, discontinuations or dropouts, and an overall evaluation.

**12. Ethical considerations**

**(1) Ethical guidelines and laws and regulations to be observed**

All persons involved in this research shall read and understand the contents of the "Declaration of Helsinki of the World Medical Association" and the "Ethical Guidelines for Life Sciences and Medical Research Involving Human Subjects" (MEXT, MHLW, METI), which all medical research involving human subjects must comply with, and shall conduct the research in full compliance with them.

**(2) Methods of protecting personal information**

**Type and method of anonymization**

Anonymization will be carried out by removing from the data and specimens of research subjects any description, etc., that would allow identification of specific individuals, such as names, and instead assigning a new code or number. Anonymization will be performed at the end of the research enrollment period. A correspondence list linking the research subject and this code (number) will be prepared by the University, and the personal information manager will keep it strictly confidential to prevent external leakage. Personal information such as the address of the research subject obtained for the purpose of delivery of sake lees and test meals will be deleted during the above anonymization.

**Personal Information Protection and Information Management System**

All parties involved in the research shall comply with applicable laws, regulations, and ordinances regarding the protection of the personal information of research subjects. In addition, those involved shall make their utmost efforts to protect the personal information and privacy of research subjects, and shall not divulge any personal information obtained in the course of conducting this research without justifiable reason. The same shall apply even after the person concerned has retired from his/her position.

When handling materials and other documents related to the implementation of the research, personal information of the subjects will be managed with an unrelated number, and sufficient consideration will be given to protecting the confidentiality of the subjects. Personal information and anonymized correspondence will be kept separately and stored in a locked desk, and will be strictly controlled to prevent leakage, theft, loss, etc. Yuri Nogami (technical assistant) is assigned as the personal information manager.

When publishing research results at academic conferences, etc., care will be taken to ensure that individuals cannot be identified, and anonymity will be protected. After the discontinuation or termination of the research, the electronic data and experimental/observational notes related to the research shall be stored for 10 years, and other research data, etc. shall be stored for 5 years from the latest of the conference presentation or publication of the paper.

**13. Procedures for obtaining informed consent**

The principal investigator or a co-investigator will give the research subjects a written explanation of consent that has been approved in advance by the Ethics Review Committee, provide sufficient explanation in writing and orally, and obtain consent in writing from the research subjects to participate in the research of their own free will as to whether or not they will participate in the research. This research will not involve persons who need to give their consent in lieu of others or persons under 16 years of age.

**14. Cost burden incurred by research subjects**

Transportation costs incurred while participating in the study will be borne by the research subject. There will be no other financial burden on the research subjects.

**Funding and Conflicts of Interest Related to the Study**

This research is supported by the Japan Agency for Medical Research and Development (AMED) Research Project for Quality Improvement of Medical Care and Collection of Scientific Evidence for "Integrative Medicine" (Project No.: 21lk0310074h0001) in FY2021 and by the Kanazawa University Hospital Publicly Invited Research for Clinical Research, etc. (Seeds B (Basic)) in FY2021. In planning, conducting, and reporting this research, we confirm that there are no "possible conflicts of interest" that could affect the results and interpretation of the research, and that the conduct of the research will not damage the rights and interests of the research subjects. In addition, the person in charge of the research shall declare the necessary information to the Kanazawa University Clinical Research Conflict of Interest Management Committee in accordance with the "Kanazawa University Clinical Research Conflict of Interest Management Policy," and shall obtain its review and approval.

**16. Changes to the implementation plan**

If, as the research progresses, it becomes necessary to change the plan in terms of research content or research organization and duration, the changes will be made after obtaining the approval of the Medical Ethics Review Committee.

**17. Samples and information**

**(1) Types, storage, recording, and destruction of samples and information**

**A. Samples obtained from the human body**

☐ N/A

☒ Yes

Sample types: blood, stool, urine

**Preservation and Destruction**

The principal investigator will instruct the research assistants to store the samples appropriately according to the established storage methods, and will conduct necessary management to prevent leakage, mixing, theft, loss, etc. of the samples. The blood samples will be stored frozen at the Graduate School of Nephrology, Kanazawa University for 10 years after the completion of the study. When disposing of the blood, it will be anonymized and personal information will be carefully handled.

**Secondary Use of Samples and Information**

Samples and information of research subjects obtained in this study may be used for future research that is not identified at the time consent is received. In such cases, a new research plan will be reviewed by the University's Ethics Review Committee and implemented after a separate explanation to the research subjects.

**About the person responsible for preservation**

The person responsible for preservation is Yusuke Nakade, a research assistant.

**B. Information**

☐ N/A

☒ Yes

**Type of information**

Subject background, stool test, urinalysis, blood test, body composition measurement, information on defecation, information on dietary intake status

**Preservation and Destruction**

The principal investigator shall instruct the research assistants to properly store the information in accordance with the specified storage method, and shall exercise necessary control to prevent leakage, theft, loss, etc. of the information. Electronic data and experiment/observation notebooks will be stored for 10 years after the research is terminated or discontinued, or after the publication of a paper, etc., and other research data, etc., will be stored for 5 years and then destroyed.

**Secondary Use of Samples and Information**

Samples and information of research subjects obtained in this study may be used for future research that is not identified at the time consent is received. In such a case, the new research plan will be reviewed by the University's Ethics Review Committee and implemented after a separate explanation to the research subjects.

**About the person responsible for preservation**

The information will be kept by the principal investigator, Kisato Tokumaru.

**(2) Records of transfer of samples and information to and from other institutions**

When providing samples and information to other institutions (including provision through partial outsourcing of operations)

☒ N/A

☐ Yes

(1) How to prepare the record of provision

Be sure to consult with the recipient before checking on the provision of the product.

- This research plan shall be used as the record of provision, and any changes shall be handled by a change request.

(In the above cases, be sure to describe the purpose of provision, etc. in the explanatory document.)

- Use the optional form* as the record of provision and respond with "Other Report" or "Implementation Status Report".

*In this case, attach the form to be reported.

*Refer to the form on the MHLW website.

http://www.mhlw.go.jp/stf/seisakunitsuite/bunya/hokabunya/kenkyujigyou/i-kenkyu/

- At the time of a new application, this research protocol shall be used as the record of provision, but thereafter, the response shall be as in 2).
- Others (Specifically: e.g., "Agreement on provision (MTA (material transfer agreement), DTA (data transfer agreement), etc.)　)

(2) Method of storing records of provision

　Storage location for records of provision:

(3) Name of the organization to which the information is provided:

(4) The name of the person responsible for the recipient:

(5) Items of sample/information to be provided:

When receiving samples and information from other institutions

☒ N/A

☐ Yes

(1) How to prepare the record of provision

- This research plan shall be used as the record of provision, and any changes shall be handled by a change request.

(In the above cases, be sure to describe the purpose of provision, etc. in the explanatory document.)

- Use the optional form* as the record of provision and respond with "Other Report" or "Implementation Status Report".

*Please attach the form to be reported at this time.

*Refer to the form on the MHLW website.

http://www.mhlw.go.jp/stf/seisakunitsuite/bunya/hokabunya/kenkyujigyou/i-kenkyu/

- At the time of a new application, this research protocol shall be the record of provision, but thereafter, the response shall be as in 2).

*Please attach the form to be reported at this time.

- Others (Specifically: e.g., "Agreement on provision (MTA (material transfer agreement), DTA (data transfer agreement), etc.)　)

(3) Method of storing records of provision

Storage location for records of provision:

(4) Name of the organization providing the service:

(5) Name of the person responsible for the provider:

(6) The method of informed consent of the donor:

(7) Disclosure of information to research subjects of the donor:

(8) Items of sample/information to be provided:

(9) How to manage the correspondence table of providers:

**18. Report to the head of the department**

☒ Adverse event reports (as needed)

☒ Reports of significant deviations from the research protocol (as needed)

☒ Implementation status report (once a year)

☒ End report (at the end of the study)

☐ Other

**19. Attribution of research results and publication of results**

Intellectual property rights may arise as a result of this research. Such rights belong to the government, research institutions, cooperative research organizations including private companies, and research workers, etc. The sample donors do not have these intellectual property rights.

**20. Research implementation system**

Principal Investigators and Research Assignees at the University

Principal Investigator:

Toshiaki Tokumaru (Kanazawa University Hospital, Kanazawa, Japan)

Research Collaborator:

Yusuke Nakade (Deputy Chief Clinical Laboratory Technologist, Department of Laboratory Medicine, Kanazawa University Hospital)

Yasunori Iwata (Kanazawa University Hospital, Division of Infection Control, Department of Infection Control)

Tadashi Toyama (Director, Division of Biostatistics, Center for Advanced Medical Research and Development, Kanazawa University Hospital, Kanazawa, Japan)

Hisayuki Ogura (Project Assistant Professor, Center for Advanced Medical Research and Development, Kanazawa University Hospital)

**21. Literature**

1. Ramezani A, et al. Role of the Gut Microbiome in Uremia: A Potential Therapeutic Target. Am J Kidney Dis. 2016 Mar;67(3):483-98.
2. Wong J, et al. Expansion of urease- and uricase-containing, indole- and p-cresol-forming and contraction of short-chain fatty acid-producing intestinal microbiota in ESRD. Am Nephrol 2014;39(3):230-237.
3. Vaziri ND, et al. Chronic kidney disease alters intestinal microbial flora. Kidney Int 2013 Feb;83(2):308-15.
4. Anders HJ, et al. The intestinal microbiota, a leaky gut, and abnormal immunity in kidney diseaseKidney Int. 2013 Jun;83(6):1010-6.
5. Abe, T., et al. Chronic kidney disease and microbiota. journal of enterobacteriology 32:15-23, 2018.
6. Guida B, et al. Effect of short-term synbiotic treatment on plasma p-cresol levels in patients with chronic renal failure: a randomized clinical trial. Nutr Metab Cardiovasc Dis. 2014 Sep;24(9):1043-9.
7. Sadao Mori et al. Effects of amazake made with sakekasu and rice malt on intestinal microbiota: A randomized, placebo-controlled, double-blind, crossover study. Pharmacology and Therapeutics Vol. 48, No. 7: 1187-1193, 2020.
8. Watanabe, Toshiro. Ingredients of "Sake Lees" Contributing to Health and Beauty. Journal of the Brewers Association of Japan, Vol. 107, No. 5; 282-291, 2012.
9. Nakade Y, et al. Gut microbiota-derived D-serine protects against acute kidney injuryJCI Insight 2018 Oct 18;3(20):e97957.
10. Rossi M, et al. Synbiotics Easing Renal Failure by Improving Gut Microbiology (SYNERGY): A Randomized Trial. Clin J Am Soc Nephrol. 2016 Feb 5;11(2):223- 31.
11. Ajimura, T. et al. Diagnosis and treatment of chronic constipation. Japanese Journal of Colorectal Diseases 72: 583-599, 2019.
12. Lewis SJ, Heaton KW. Stool form scale as a useful guide to intestinal transit time. Scand J Gastroenterol. 1997 Sep;32(9):920-4.
13. KIRA Izumi. Reliability and validity of the Japanese version of The Patient Assessment of Constipation Quality of Life Questionnaire. Journal of the Japanese Society of Nursing Research, Vol. 36, No. 2; 119-127. 2013.
14. Takahashi, Keiko. Development of a Food Intake Status Questionnaire for Estimating Intake of Nutrients and Food Groups (Brief Survey Method). Journal of Nutrition Vol. 61 No. 3;161-169. 2003.

When a portion of the research work is outsourced, the nature of the work and the method of supervision of the outsourced party.

☒ Not entrusted

☐ Commissioning

**23. Monitoring**

☒ Not applicable (because it is not invasive)

☐ Yes

**24. Auditing**

☒ N/A

☐ Yes

**25. Consultation service**

The Nutritional Management Department of Kanazawa University Hospital will serve as the point of contact for consultations, etc., with research subjects, etc., and their concerned parties.

Address: 13-1 Takara-machi, Kanazawa 920-8641

Department of Nutritional Management, Kanazawa University Hospital

Phone: 076-265-2087 (ext. 2087)

FAX: 076-234-4331

**Other precautions**

　No special note
