# Supplementary material for: Effects of sake lees intake on fecal uremic toxins, plasma D-alanine, constipation, and gut microbiome in healthy adults: A single-arm clinical trial
Source: PLoS One. 2025 Jun 3;20(6):e0325482. doi: 10.1371/journal.pone.0325482 (PMC12133169; doi:10.1371/journal.pone.0325482)
Supplement: S2 Protocol — (DOCX) [file pone.0325482.s007.docx]

２０２１年１０月８日作成　Ver. 1.0

**研究計画書**

**１．課題名**

慢性便秘患者に対する酒粕摂取の腸内細菌叢および代謝産物の変化に関する研究

**２．研究の概要・目的・意義**

**概要**

慢性便秘患者に対する酒粕摂取による腸内環境への有用性を単群 (2用量) による介入試験で評価する(**図**)。主要評価項目は便中の尿毒症物質とし、副次評価項目は便中の菌叢、短鎖脂肪酸などとする。

（**図**）研究デザインの概要

**目的**

本研究計画書に記載する研究（以下、本研究）は、慢性便秘患者における酒粕摂取量別・摂取期間別の便中の代謝産物および菌叢の変化を評価することを目的とするパイロット研究 である。本研究で得られた結果から、慢性腎臓病 (CKD) 患者を対象とした小規模ランダム化比較試験 (RCT) における酒粕摂取量と摂取期間を設定する。

**背景**

CKD患者は、腸内細菌叢が乱れている状態であるディスバイオーシス (DysB) が存在することが知られている^1)^。CKD患者では血中の尿素や尿酸が上昇して腸管に移行することや、薬物療法、食事制限による食物繊維不足などによりDysBを生じる。これらにより、腸管上皮のエネルギー源となり、腸管の免疫制御に関与する短鎖脂肪酸を産生する有益な菌種は減少し^2)^、逆にインドールやクレゾールなどの尿毒症物質を産生する有害な菌種は増加する^3)^。すなわち、DysBによって産生される代謝産物が変化し、CKDが進展する悪循環が形成されている可能性がある^4)^。例えば、短鎖脂肪酸の不足は腸上皮バリアの脆弱化を引き起こし、本来は腸管内にとどまるべき病原菌や尿毒症物質が腸上皮を通過して血中へ移行し、慢性炎症などを介し腎障害が進行する可能性がある^5)^。

このことにより、CKD患者における腸内細菌叢および代謝産物の制御は、CKD進展予防において重要である。そのために、腸内細菌叢および代謝産物の制御に関与する因子を明らかにする必要がある。

腸内細菌叢および代謝産物に関与する因子として、食事が挙げられる。DysBの一般的な治療として、プロバイオティクス (有益菌の摂取、ProB) ・プレバイオティクス (食物繊維などの摂取、PreB) と呼ばれる食事療法が行われ、CKD患者においてProB・PreBが試験的に行われている。例えば、CKD患者を対象としたProB・PreBにより、血中尿毒症物質 (p-クレシル硫酸) 濃度が減少したと報告されている^6)^。よって、CKD患者に適したProB・PreBは、有用なDysB治療となる可能性がある。

酒粕は有益菌や食物繊維を含み、CKD患者に適したProB・PreB両者の働きを有する。加えて、CKD患者において制限対象となるリンやカリウムの含有量が少なく、食塩量も少ない。その効果として、便秘のある者を対象とした研究では、4週間の甘酒190g (酒粕25g相当)の摂取により腸内細菌叢が改善する報告や^7)^、3週間の酒粕50gの摂取により便秘を改善する効果が報告されている^8)^。

D-アミノ酸の一種であるD-セリンおよびD-アラニンは、腎保護効果を有することを研究分担者らが確認している^9, 論文投稿中)^。酒粕などの発酵食品はD-アミノ酸を含み、酒粕からD-アミノ酸を直接摂取することで、腎保護効果が得られる可能性がある。しかし、酒粕摂取よる便中および血中のD-セリン、D-アラニンの変化をみた研究は確認ができない。また、酒粕摂取と便中菌叢を見た既報の研究において、尿毒症物質や短鎖脂肪酸などの代謝産物に関わる便中菌叢の変化をみた研究は検索した限り存在せず、酒粕摂取の有用性を示す評価指標のデータが不足している。

そこで本研究では、CKDのない慢性便秘患者における酒粕摂取量別・摂取期間別の便中の代謝産物および菌叢の変化を評価することを目的とする。本研究で得られた結果から、CKD患者を対象とした小規模RCTにおける酒粕摂取量と摂取期間を設定する。

**３．研究の科学的合理性と根拠**

**研究概要と主要評価項目**

本研究の対象は慢性便秘患者とし、介入は酒粕25 g/日あるいは50 g/日の摂取とする。対照は介入前とし、主要評価項目は2週後、4週後、6週後の便中の尿毒症物質 (インドールおよびクレゾール) 濃度の変化率とする。研究デザインは、単群 (2用量) の前後比較試験とする。

**研究デザインの妥当性**

本研究の対象者はCKDのない慢性便秘患者とする。これまでCKD患者に対する酒粕を用いた研究は検索した限り存在せず、慢性便秘患者を対象とした研究がいくつかある。酒粕摂取により有益菌とされる特定の菌種が増加する報告や^7)^、便秘を改善する効果が報告されている^8)^。酒粕摂取により腸内細菌叢および尿毒症物質の変化が推測され、そしてその変化は腎機能が低下していない者でも確認できると予想されることから、CKDのない慢性便秘患者を対象とする。

介入は、酒粕25 g/日あるいは50 g/日の6週間の摂取とする。既報において、便秘のある者を対象とした研究では、4週間の甘酒190 g (酒粕25 g相当)の摂取により腸内細菌叢が改善する報告や^7)^、3週間の酒粕50 gの摂取により便秘が改善する効果が示されている^8)^。またCKD 患者を対象とした研究では、6週間のシンバイオティクスのサプリメントの摂取により血中の尿毒症物質濃度が減少したと報告されている^10)^。これらから、本研究の介入は酒粕25 g/日あるいは50 g/日の計6週間の摂取とし、得られた結果からCKD 患者を対象とした小規模RCT における酒粕摂取量と摂取期間を設定することとする。

アウトカムの評価方法は、比較対照者をおかない前後比較研究とする。本研究は、CKD 患者を対象とした小規模RCTの酒粕摂取量と摂取期間を設定する根拠を得るために実施する研究である。根拠として得たい便中の尿毒症物質の変化は、介入前後の比較で確認が可能であることから、比較対象群を置かない、単群 (2用量) の前後比較研究とする。評価は介入時、2週後、4週後、6週後の計4回実施し、小規模RCTの酒粕摂取量と摂取期間を設定する。

**研究目的と主要評価項目との合致**

本研究の目的は、最終目標であるCKD患者に対する酒粕摂取による血中の尿毒症物質の変化を観察するに際し、その過程である便中の尿毒症物質の変化を確認することである。血中の尿毒症物質の変化について、ProB・PreBサプリメントを用いた血中の尿毒症物質の変化をみた研究はいくつかある。例えばCKDステージ3から4の患者を対象にProB・PreBサプリメント摂取と血中の尿毒症物質 (p-クレシル硫酸) への影響をみた研究において、2週後および4週後のp-クレシル硫酸が有意に減少したと報告されている^6)^。またCKDステージ4から5の非透析患者を対象とした同様の研究においても、6週後のp-クレシル硫酸が減少したと示されている^10)^。これら結果は、ProB・PreBにより腸内細菌叢と腸内の代謝産物が変化し、それによって血中の尿毒症物質が減少した機序が有力である。この機序解明には、酒粕摂取による便中の尿毒症物質の確認が必要であることから、主要評価項目の評価を行うことで研究目的が達成できる。なお、腎機能が正常である健常者においては、酒粕摂取前後で血中の尿毒症物質は変化が生じないと予想されるため、本研究では血中の尿毒症物質の評価は実施しない。

**４．研究対象者及び選定方針**

**（１）適格基準**

1. 承認日から2022年1月までに、便秘の診断基準^11)^を満たす健常者

1．「便秘症」の診断基準

以下の 6 項目のうち、2 項目以上を満たす

1. 排便の 4 分の 1 超の頻度で、強くいきむ必要がある．
2. 排便の 4 分の 1 超の頻度で、兎糞状便または硬便（ブリストル便形状スケール でタイプ 1 か 2 ）である．
3. 排便の 4 分の 1 超の頻度で、残便感を感じる．
4. 排便の 4 分の 1 超の頻度で、直腸肛門の閉塞感や排便困難感がある．
5. 排便の 4 分の 1 超の頻度で、用手的な排便介助が必要である（摘便・会陰部圧迫など）．
6. 自発的な排便回数が、週に 3 回未満である．

2．「慢性」の診断基準

6 ヵ月以上前から症状があり，最近 3 ヵ月間は上記の基準を満たしていること

1. 本研究の参加に関して同意が文書で得られる者
2. 同意取得時の年齢が20歳以上の者

**（２）除外基準**

1. アルコール不耐症の者
2. 研究参加時から過去4週間以内に抗菌薬使用歴のある者
3. 下痢や便秘が副作用にある薬剤を服用中の者
4. その他、医師の判断により対象として不適当と判断された者

**５．目標数と研究実施期間**

**（１）目標数**

全体目標数　8例

**（２）研究期間**

研究期間：　　　　　　　　承認日～2023年3月31日

登録期間：　　　　　　　　承認日～2022年1月31日

観察期間（追跡期間）： 登録終了後から3ヶ月間

解析期間：　　　　　　　　承認日～2023年3月31日

**６．研究方法**

**（１）研究のデザイン**

単群 (2用量) による前後比較試験 (単施設)

**（２）研究の方法**

**研究参加者の募集**

　金沢大学内での掲示板やメーリングリストを通じて金沢大学職員に通知し、職員の家族を含め参加者を募る (本研究の関係者およびその家族は除く) 。

**研究対象者の割付**

研究対象者への酒粕摂取量および摂取期間の割付は、研究代表者が行う。研究対象者が登録されるごとに、酒粕摂取量および摂取期間を番号順に割付を行う。

**研究の手順**


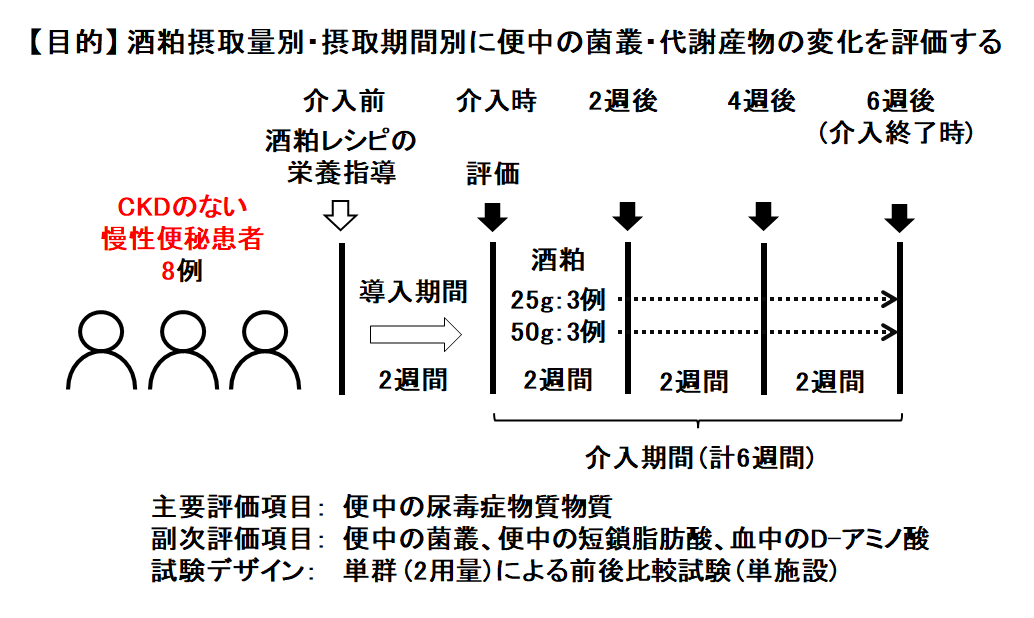


1. 介入前に、独自に作成した酒粕レシピ(別紙1)用いた栄養指導を実施する
2. 酒粕25g/日あるいは酒粕50g/日を6週間摂取する
3. 酒粕は、金沢大学が販売する清酒 「角間の里」 の酒粕を用いる。介入条件を揃えるため、同一ロットの酒粕を参加者へ無償で提供する
4. 酒粕は冷蔵庫等にて5℃程度で保存する
5. 酒粕の調理方法は、酒粕レシピ集の中から参加者が選択することができる
6. アウトカム項目の評価は、介入時、2週後、4週後、6週後に実施する
7. 酒粕の摂取状況、排便状況は記録表に毎日記録する
8. 酒粕摂取のタイミングおよび摂取回数は設定しない。例えば、酒粕25gを1回の食事でまとめて摂取しても、3回の食事で分割して摂取しても可とする
9. 導入期間 （2週間） および介入期間 (6週間) は、乳酸菌製剤および腸内環境改善を表示する特定保健用食品、機能性表示食品の摂取を禁止する。酒粕以外の味噌やヨーグルトなどの発酵食品 (一般食品) の摂取は可とする
10. 評価前日の夕食は同一 (栄養価が管理された冷凍の宅配弁当) とする
11. 2週後、4週後、6週後の評価当日の朝食は酒粕を含んだ同一の献立を摂取する

**試料の取得**

次の方法で試料を得る。参加者に対し、別に作成する検便の方法や提出場所などを記した行動予定表を用い、同意取得時に説明を行う。

1. 介入時、酒粕摂取の2週後、4週後、6週後に検便を実施し、便中の尿毒症物質濃度、短鎖脂肪酸濃度、便中菌叢を得る
2. 介入時および6週後に検尿を実施し、尿中のpHを得る
3. 検便および検尿は、参加者の自宅にて所定のキットを用い採取する
4. 介入時、酒粕摂取の2週後、4週後、6週後に採血を実施し、血中のD-アミノ酸濃度を得る
5. 独自に作成した記録表(別紙2)を用いて酒粕摂取状況を被検者が毎日記録する
6. ブリストルスケール^12)^(別紙3)を用いて排便回数および便性状を被検者が毎日記録する
7. 介入開始2週間前および介入終了時 （6週後） に妥当性が確認されている食事評価法を実施し、食物摂取頻度、摂取栄養素量を評価する

※④は採血時に軽微な侵襲を伴う。①②③⑤⑥は検便、検尿およびアンケートによる取得のため、身体的な侵襲は加わらない。

**研究の実施場所**

診察、採血、栄養指導の実施は、金沢大学大学院腎臓内科学研究室内とする

体組成測定の実施は、金沢大学附属病院栄養相談室内とする

**データ解析方法**

主要評価項目である便中の尿毒症物質濃度は、介入前後の値を分散分析およびダネット検定を用い、両側検定、有意水準5%にて解析する。

**研究結果の開示**

同意取得の際、参加者に研究結果の開示の希望の意思確認を行い、開示の希望があった場合は、検便、検尿、血液検査の結果を開示する。開示の方法は、研究参加者に対し書面で開示する。ただし、研究の途中で開示希望の取り消しがあった場合は、開示は行わない。

**７．観察・検査・報告項目**

観察・検査項目は次のとおりとする。

**被検者背景**

年齢 （生年） 、性別、身長、体重、BMI

**便検査**

尿毒症物質 (インドール、 p-クレゾール) 、 菌叢、短鎖脂肪酸 (酢酸、 乳酸、 プロピオン酸、 酪酸) 、pH

**尿検査**

　pH

**血液検査**

D-セリン、 D-アラニン

**排便回数、便性状**

ブリストルスケール、便秘と生活の質のスコア (PAC-QOL)^13)^ (別紙4)

**食物摂取頻度および摂取栄養素量評価**

食物摂取頻度調査 (FFQg)^14)^ (別紙5)、酒粕摂取の遵守状況

**体組成測定**

骨格筋量、体幹筋量、四肢筋量、体脂肪率、体脂肪量、 体細胞量、細胞外水分比

**観察・検査のスケジュール**

検査期間の幅は、基準日から前後5日以内とする。

| **基準日** | **介入前 (-2週間)** | **介入時** | **2週後** | **4週後** | **6週後 （介入終了時)** |
| --- | --- | --- | --- | --- | --- |
| **Visit** | **1** | **2** | **3** | **4** | **5** |
| **医師の診察** | **●** |  |  |  |  |
| **同意取得** | **●** |  |  |  |  |
| **栄養指導** | **●** | **●** | **●** | **●** | **●** |
| **食物摂取頻度調査** | **●** |  |  |  | **●** |
| **便秘とQOLスコア** | **●** | **●** | **●** | **●** | **●** |
| **体組成測定** |  | **●** | **●** | **●** | **●** |
| **便検査** |  | **●** | **●** | **●** | **●** |
| **尿検査** |  | **●** |  |  | **●** |
| **血液検査** |  | **●** | **●** | **●** | **●** |
| **酒粕摂取状況** |  |  | **毎日** | | |
| **排便回数、便性状** |  |  | **毎日** | | |

**８．有害事象の評価と報告**

**（１）有害事象の定義と報告方法**

■該当なし

□該当あり

本研究では、介入前後に採血を実施するため軽微な侵襲を伴う。採血時に研究対象者の状態を十分に観察し、不調があれば採血を中止する。

酒粕は日常的に摂取する食品であり摂取による有害事象の発生は想定されないが、好ましくない体調変化を生じた場合は、窓口を通じて研究分担医師に相談の上対応する。医師により研究の継続が困難と判断された場合は酒粕摂取を中止し、部局長へ報告する。

**（２）研究対象者に生じる負担並びに予測されるリスク及び利益、これらの総合的評価並びに当該負担及びリスクを最小化する対策**

**①予測される利益**

本研究に参加することによる研究対象者個人への直接的な利益は生じない。研究成果により医療の進歩に貢献できる可能性がある。謝礼として、研究終了時に本学規程に基づきQUOカード(上限20,000円分)を進呈する。

使用する酒粕は、介入条件を揃えるため同一製品・同一ロットを用いる必要があることから、酒粕を研究対象者へ無償で提供する。

**②予測されるリスクと不利益**

本研究では、介入前後に採血を実施するため軽微な侵襲を伴う。採血時に研究対象者の状態を十分に観察し、不調があれば採血を中止する。

酒粕は微量のアルコールを含むため、アルコール揮発が不十分な酒粕摂取後の車の運転は、飲酒運転に該当する可能性がある。プロトコールにそった調理法を遵守してアルコールを十分に揮発させた酒粕を使用することを、介入時の栄養指導で重点的に説明する。

**９．評価項目**

**（１）主要評価項目**

便中の尿毒症物質 (インドールおよびクレゾール)　濃度の変化率

**（２）副次評価項目**

便中の主要菌種の占める割合の変化率

便中の短鎖脂肪酸 (酢酸、 乳酸、 プロピオン酸、 酪酸) 濃度

便のpH、 便性状、 排便回数

血中のD-アミノ酸濃度

排便回数および便性状、便秘と生活の質のスコア (PAC-QOL)

食物摂取頻度、摂取栄養素量、酒粕の遵守割合

**１０．統計的事項**

**症例数設計**

実施可能性に基づき8例と設定した。

**１１．症例報告書の記入と報告**

症例報告書には研究参加者の基本属性、介入期間の各種検査データ、担当医師の所見、中止・脱落、総合評価を記入する。

**１２．倫理的配慮**

**（１）遵守する倫理指針や法令**

本研究に携わるすべての者は、人を対象とする全ての医学研究が準拠すべき「世界医師会ヘルシンキ宣言」及び「人を対象とする生命科学・医学系研究に関する倫理指針」（文部科学省・厚生労働省・経済産業省）の内容を熟読し理解した上で遵守し、研究を施行する。

**（２）個人情報の保護の方法**

**匿名化の種類及び方法**

研究対象者のデータや検体から氏名等の特定の個人を識別することができることとなる記述等を削り、代わりに新しく符号又は番号をつけて匿名化を行う。匿名化は、研究登録期間の終了時に実施する。研究対象者とこの符号（番号）を結びつける対応表を本学で作成し、個人情報管理者は外部の漏れないように厳重に保管する。酒粕や検査食の配送を目的に入手した研究対象者の住所等の個人情報は、上記の匿名化の際に削除する。

**個人情報保護および情報管理体制**

研究に関わる関係者は、研究対象者の個人情報保護について、適用される法令、条例を遵守する。 また関係者は、研究対象者の個人情報およびプライバシー保護に最大限の努力を払い、本研究を行う上で知り得た個人情報を正当な理由なく漏らしてはいけない。関係者がその職を退いた後も同様とする。

研究実施に係る資料等を取り扱う際は、被験者の個人情報等は無関係の番号を付して管理し、被験者の秘密保護に十分配慮する。個人情報および匿名化した場合の対応表は別に保管し、それぞれ施錠された机に保管し，漏洩・盗難・紛失等が起こらないように厳重に管理する。個人情報管理者として、野上有里 (技術補佐員) を置く。

学会などで研究結果を公表する際には個人が特定できないように配慮し、匿名性を守る。研究の中止又は終了後、学会発表、論文発表のうち、最も遅い時期から、研究に関する電子データ及び実験・観察ノートは10年、その他研究データ等は5年保存する。

**１３．インフォームド・コンセントを受けるための手続きについて**

研究責任者又は分担研究者は、事前に倫理審査委員会で承認の得られた同意説明文書を研究対象者に渡し、文書及び口頭による十分な説明を行い、研究に参加するかどうかについて、研究対象者の自由意思による研究参加の同意を文書で得る。なお本研究は代諾が必要な者、16歳未満の者を研究対象者としない。

**１４．研究対象者に生じる費用負担について**

研究に参加中に発生する交通費は、研究対象者が負担する。それ以外の研究対象者の経済的負担はない。

**１５．本研究に係る資金ならびに利益相反について**

本研究は、令和3年度日本医療研究開発機構 (AMED)「統合医療」に係る医療の質向上・科学的根拠収集研究事業 (課題管理番号：21lk0310074h0001) 、2021年度金沢大学附属病院臨床研究等に係る公募研究 (シーズB(基礎)) の研究助成を得て実施する。本研究の計画・実施・報告において、研究の結果および解釈に影響を及ぼすような「起こりえる利益相反」は存在しないこと、および研究の実施が研究対象者の権利・利益を損ねることがないことを確認する。また、本研究の研究担当者は、「金沢大学臨床研究利益相反マネージメントポリシー」に従い、金沢大学臨床研究利益相反マネージメント委員会に必要事項を申告し、その審査と承認を得るものとする。

**１６．実施計画の変更について**

研究の進捗にともない、研究内容及び研究組織・期間などに計画の変更の必要が生じた場合は、医学倫理審査委員会の承認を得て、変更を行う。

**１７．試料・情報について**

**（１）試料・情報の種類、保存、記録、破棄について**

A．人体から取得した試料

□該当なし

■該当あり

試料の種類： 血液、便、尿

**保存・破棄について**

研究責任者は、定められた保管方法に従って研究分担者等が適切に保管するよう指導し、試料の漏えい、混交、盗難、紛失等が起こらないよう必要な管理を行う。採取した血液は、研究終了後10年まで金沢大学大学院腎臓内科学にて冷凍保管する。廃棄する際は、匿名化し個人情報に注意して行う。

**試料及び情報の二次利用について**

本研究で得られた研究対象者の試料・情報は、同意を受ける時点では特定されない将来の研究のために用いる可能性がある。その場合には、新たな研究計画について本学倫理審査委員会の審査を受けたうえで、別途研究対象者に説明した上で実施する。

**保存の責任者について**

保存の責任者は研究分担者 中出 祐介 とする。

B．情報

□該当なし

■該当あり

**情報の種類**

対象者背景、便検査、尿検査、血液検査、体組成測定、排便に関する情報、食事摂取状況に関する情報

**保存・破棄について**

研究責任者は、定められた保管方法に従って研究分担者等が適切に保管するよう指導し、情報の漏えい、盗難、紛失等が起こらないよう必要な管理を行う。電子データ及び実験・観察ノートは研究終了若しくは中断または、論文等が発表されてから遅い時期から10年間、その他の研究データ等は5年間保存した後、破棄する。

**試料及び情報の二次利用について**

本研究で得られた研究対象者の試料・情報は、同意を受ける時点では特定されない将来の研究のために用いる可能性がある。その場合には、新たな研究計画について本学倫理審査委員会の審査を受けたうえで、別途研究対象者に説明した上で実施する。

**保存の責任者について**

情報は研究責任者 徳丸 季聡 が保管する。

（２）試料・情報の他機関との授受の記録について

【他機関に試料・情報を提供する場合（業務の一部委託による提供を含む）】

■該当なし

□該当あり

①提供記録の作成方法

＊提供については、必ず提供先と相談の上、チェックすること。

□1）本研究計画書を提供記録とし、変更時は変更申請で対応する。

（上記の場合は、必ず説明文書に提供目的等を記載のこと）

□2）任意様式※を提供記録とし、「その他報告」か「実施状況報告」で対応する。

＊この際は報告する様式を添付すること

※厚労省HPの様式を参考にすること。

http://www.mhlw.go.jp/stf/seisakunitsuite/bunya/hokabunya/kenkyujigyou/i-kenkyu/

□3）新規申請時は、本研究計画書を提供記録とするが、その後は2）の対応とする。

＊この際は報告する様式を添付してください

□4）その他（具体的に：例：「提供に関する契約書（ＭＴＡ（material transfer agreement）、ＤＴＡ（data transfer agreement）等）」を用いる。　）

②提供記録の保管方法

　・提供記録の保管場所：

③提供先の機関名称：

④提供先の責任者名：

⑤提供する試料・情報の項目：

【他機関から試料・情報の提供を受ける場合】

■該当なし

□該当あり

①提供記録の作成方法

＊提供については、必ず提供元と相談の上、チェックしてください

□1）本研究計画書を提供記録とし、変更時は変更申請で対応する。

（上記の場合は、必ず説明文書に提供目的等を記載のこと）

□2）任意様式※を提供記録とし、「その他報告」か「実施状況報告」で対応する。

＊この際は報告する様式を添付してください

※厚労省HPの様式を参考にすること。

http://www.mhlw.go.jp/stf/seisakunitsuite/bunya/hokabunya/kenkyujigyou/i-kenkyu/

□3）新規申請時は、本研究計画書を提供記録とするが、その後は2）の対応とする。

＊この際は報告する様式を添付してください

□4）その他（具体的に：例：「提供に関する契約書（ＭＴＡ（material transfer agreement）、ＤＴＡ（data transfer agreement）等）」を用いる。　）

③提供記録の保管方法

・提供記録の保管場所：

④提供元の機関名称：

⑤提供元の責任者名：

⑥提供元のインフォームド・コンセントの方法：

⑦提供元の研究対象者への情報公開：

⑧提供を受ける試料・情報の項目：

⑨提供元の対応表の管理方法：

**１８．部局長への報告**

■　有害事象報告（随時）

■　研究計画書からの重大な逸脱に関する報告（随時）

■　実施状況報告（年１回）

■　終了報告（研究終了時）

□　その他　（　　　　　）

**１９．研究成果の帰属と結果の公表**

本研究の結果として知的財産権が生じる可能性がある。その権利は国、研究機関、民間企業を含む共同研究機関及び研究従事者などに属し、試料提供者にはこの知的財産権は属さない。

**２０．研究実施体制**

本学における研究責任者及び研究分担者

研究責任者　徳丸 季聡 （金沢大学附属病院 栄養管理部 栄養管理室長）

研究分担者　中出 祐介 （金沢大学附属病院 検査部 副臨床検査技師長）

研究分担者　岩田 恭宜 （金沢大学附属病院 感染制御部 感染制御部長）

研究分担者　遠山　直志 （金沢大学附属病院 先端医療開発センター 生物統計部門長）

研究分担者　小倉　央行 （金沢大学附属病院 先端医療開発センター 特任助教）

**２１．文献**

1. Ramezani A, et al. Role of the Gut Microbiome in Uremia: A Potential Therapeutic Target. Am J Kidney Dis. 2016 Mar;67(3):483-98.
2. Wong J, et al. Expansion of urease- and uricase-containing, indole- and p-cresol-forming and contraction of short-chain fatty acid-producing intestinal microbiota in ESRD. Am Nephrol 2014;39(3):230-237.
3. Vaziri ND, et al. Chronic kidney disease alters intestinal microbial flora. Kidney Int 2013 Feb;83(2):308-15.
4. Anders HJ, et al. The intestinal microbiota, a leaky gut, and abnormal immunity in kidney diseaseKidney Int. 2013 Jun;83(6):1010-6.
5. 阿部 高明. 慢性腎臓病とmicrobiota. 腸内細菌学雑誌32:15-23, 2018.
6. Guida B, et al. Effect of short-term synbiotic treatment on plasma p-cresol levels in patients with chronic renal failure: a randomized clinical trial. Nutr Metab Cardiovasc Dis. 2014 Sep;24(9):1043-9.
7. 森　貞夫 ほか. 酒粕と米麹を使用した甘酒の摂取による腸内細菌叢へ及ぼす効果―ランダム化プラセボ対照二重盲検クロスオーバー群間比較試験―. 薬理と治療48巻7号:1187-1193, 2020.
8. 渡辺 敏郎. 健康と美容に貢献する「酒粕」の成分. 日本醸造協会誌107巻5号;282-291, 2012.
9. Nakade Y, et al. Gut microbiota-derived D-serine protects against acute kidney injuryJCI Insight 2018 Oct 18;3(20):e97957.
10. Rossi M, et al. Synbiotics Easing Renal Failure by Improving Gut Microbiology (SYNERGY): A Randomized Trial. Clin J Am Soc Nephrol. 2016 Feb 5;11(2):223-31.
11. 味村 俊樹 ほか. 慢性便秘症の診断と治療. 日本大腸肛門病会誌　72：583-599，2019.
12. Lewis SJ, Heaton KW. Stool form scale as a useful guide to intestinal transit time. Scand J Gastroenterol. 1997 Sep;32(9):920-4.
13. 吉良 いずみ. 日本語版The Patient Assessment of Constipation Quality of Life Questionnaireの信頼性と妥当性の検討. 日本看護研究学会雑誌36巻2号;119-127. 2013.
14. 高橋 啓子. 栄養素および食品群別摂取量を推定するための食物摂取状況調査票(簡易調査法)の作成. 栄養学雑誌 Vol. 61 No.3;161-169. 2003.

**２２．研究に関する業務の一部を委託する場合の、当該業務内容及び委託先の監督方法**

■委託しない

□委託する

**２３．モニタリングについて**

■該当なし (侵襲を伴わないため)

□該当あり

**２４．監査について**

■該当なし

□該当あり

**２５．相談窓口**

研究対象者等及びその関係者からの相談等への対応窓口として、金沢大学附属病院栄養管理部が対応する。

住所　〒920-8641 金沢市宝町13-1

金沢大学附属病院栄養管理部　　担当　徳丸 季聡

　　　　　　　　　電話： 076-265-2087 （内線2087）

　FAX： 076-234-4331

・・・・・・・・・・・・・・・・・・・・・・・・・・・・・・・・・・・・・・・・・・・・・・・・・・・・・・・・・・・・・・

**その他注意事項**

　特記事項なし
