# Supplementary material for: Effects of sake lees intake on fecal uremic toxins, plasma D-alanine, constipation, and gut microbiome in healthy adults: A single-arm clinical trial
Source: PLoS One. 2025 Jun 3;20(6):e0325482. doi: 10.1371/journal.pone.0325482 (PMC12133169; doi:10.1371/journal.pone.0325482)
Supplement: S1 File — (DOCX) [file pone.0325482.s009.docx]

For measurement of the levels of phenols and indoles, 0.1 g feces was suspended with 2.5 mL of phosphate buffer including 0.4 mg/L 4-isopropylphenol as an internal standard. The sample was heated at 85°C for 15 min, mixed with 2.5 mL acetonitrile and 1 g NaCl, shaken for 30 min, and centrifuged at 1,300 ×*g* for 10 min. One milliliter of the supernatant was dehydrated and purified by a sodium sulfate drying cartridge with C18 and PSA (GL Sciences, Tokyo, Japan) and placed into a vial.

Phenols and indoles in feces were measured by gas chromatography equipped with a mass spectrometry (5977A, Agilent, CA, USA) and a capillary column (DB-WAX UI+G, 60 m × 0.25 mm × 0.25 µm, Agilent). Helium was used as the carrier gas at 1.7 mL/min. The injector temperature program was as follows: 70°C for 2 min; then 200°C/min to 240°C. The interface temperature was maintained at 230°C. The oven temperature program was as follows: 70°C for 2 min; then 25°C/min to 190°C, held for 2 min; 2°C /min to 200°C, held 1.5 min; 25°C/min to 240°C; finally 240°C, held for 8 min). The extract (0.8 µL) was injected in the splitless mode. Data acquisition was performed in the selected ion monitoring (SIM) mode for quantification.
