# Supplementary material for: Effects of sake lees intake on fecal uremic toxins, plasma D-alanine, constipation, and gut microbiome in healthy adults: A single-arm clinical trial
Source: PLoS One. 2025 Jun 3;20(6):e0325482. doi: 10.1371/journal.pone.0325482 (PMC12133169; doi:10.1371/journal.pone.0325482)
Supplement: Supplementary file 6 [file pone.0325482.s001.docx]

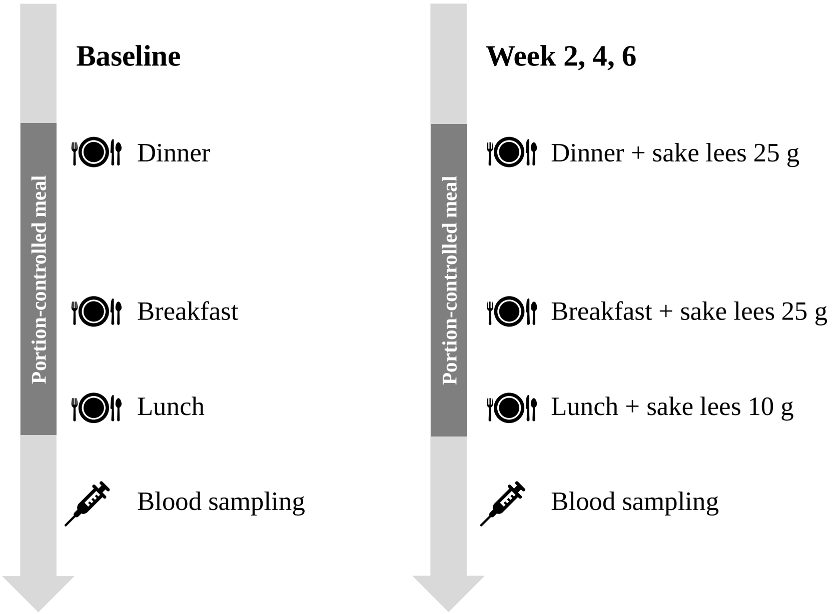
Baseline
Portion-controlled meal
Dinner
Breakfast
Lunch
Blood sampling
Week 2, 4, 6
Portion-controlled meal
Dinner + sake lees 25 g
Breakfast + sake lees 25 g
Lunch + sake lees 10 g
Blood sampling
