# Supplementary material for: Effects of sake lees intake on fecal uremic toxins, plasma D-alanine, constipation, and gut microbiome in healthy adults: A single-arm clinical trial
Source: PLoS One. 2025 Jun 3;20(6):e0325482. doi: 10.1371/journal.pone.0325482 (PMC12133169; doi:10.1371/journal.pone.0325482)

A ***Bacteroides* (genus level)**

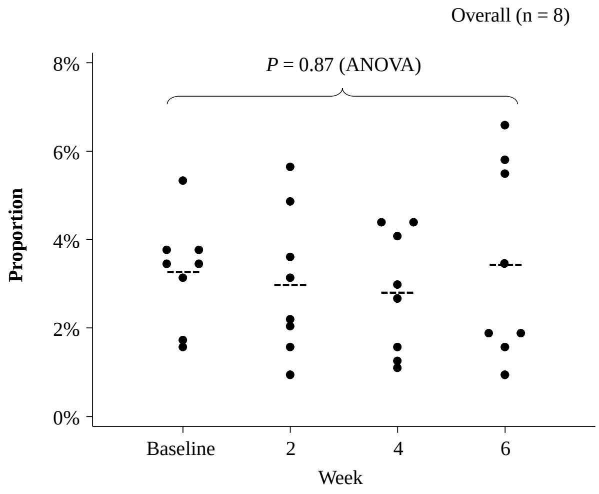

B ***Coriobacteriaceae* (family level)**

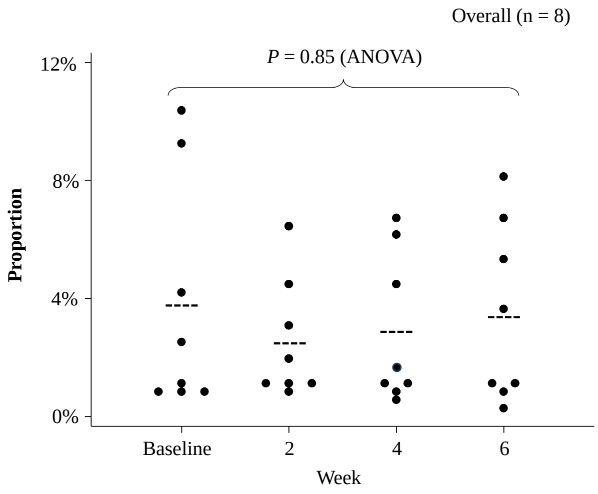

Supplement: Supplementary file 7 [file pone.0325482.s002.docx]
