# Supplementary material for: Effects of sake lees intake on fecal uremic toxins, plasma D-alanine, constipation, and gut microbiome in healthy adults: A single-arm clinical trial
Source: PLoS One. 2025 Jun 3;20(6):e0325482. doi: 10.1371/journal.pone.0325482 (PMC12133169; doi:10.1371/journal.pone.0325482)
Supplement: Supplementary file 8 [file pone.0325482.s003.docx]

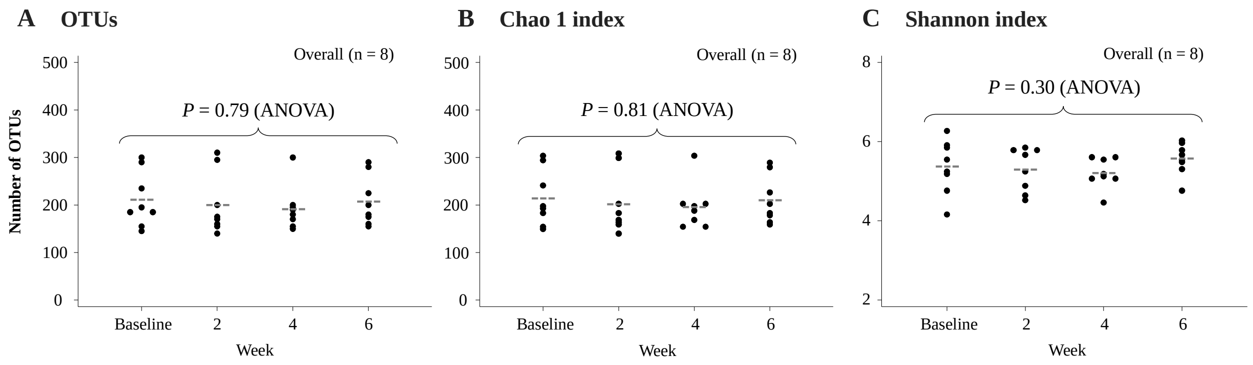
A OTUs
Overall (n = 8)
P = 0.79 (ANOVA)
Number of OTUs
500
400
300
200
100
0
Baseline
2
4
6
Week
B Chao 1 index
Overall (n = 8)
P = 0.81 (ANOVA)
500
400
300
200
100
0
Baseline
2
4
6
Week
C Shannon index
Overall (n = 8)
P = 0.30 (ANOVA)
8
6
4
2
Baseline
2
4
6
Week
