# Supplementary material for: Effects of sake lees intake on fecal uremic toxins, plasma D-alanine, constipation, and gut microbiome in healthy adults: A single-arm clinical trial
Source: PLoS One. 2025 Jun 3;20(6):e0325482. doi: 10.1371/journal.pone.0325482 (PMC12133169; doi:10.1371/journal.pone.0325482)
Supplement: Supplementary file 9 [file pone.0325482.s004.docx]

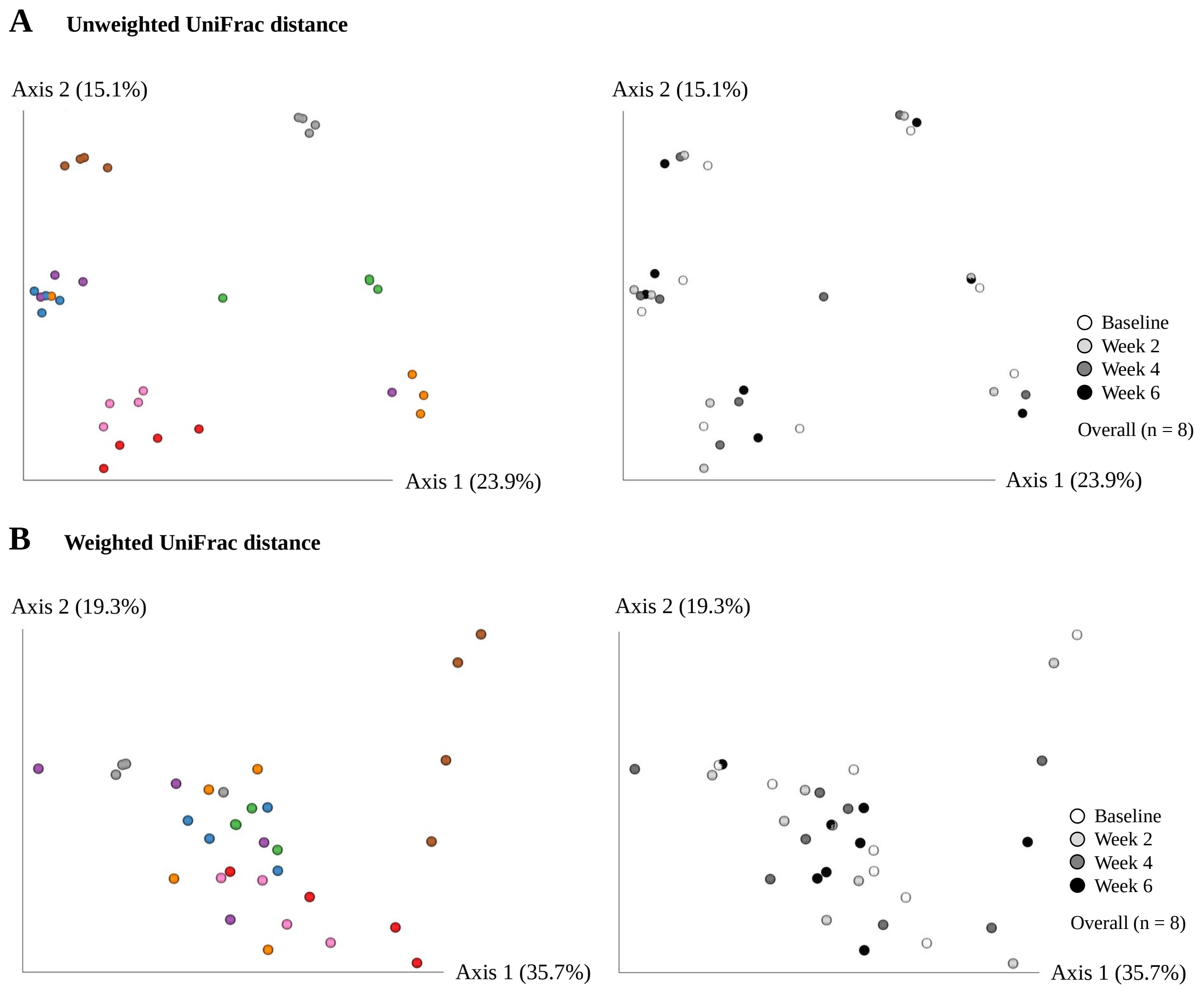
A
Unweighted UniFrac distance
Axis 2 (15.1%)
Axis 1 (23.9%)
Axis 2 (15.1%)
Axis 1 (23.9%)
Baseline
Week 2
Week 4
Week 6
Overall (n = 8)
B
Weighted UniFrac distance
Axis 2 (19.3%)
Axis 1 (35.7%)
Axis 2 (19.3%)
Axis 1 (35.7%)
Baseline
Week 2
Week 4
Week 6
Overall (n = 8)
