# Supplementary material for: Effects of sake lees intake on fecal uremic toxins, plasma D-alanine, constipation, and gut microbiome in healthy adults: A single-arm clinical trial
Source: PLoS One. 2025 Jun 3;20(6):e0325482. doi: 10.1371/journal.pone.0325482 (PMC12133169; doi:10.1371/journal.pone.0325482)
Supplement: Attachment — Submitted filename: Response to the Reviewer.docx [file pone.0325482.s011.docx]

# **Journal requirements**

**Point 1**

Please ensure that your manuscript meets PLOS ONE's style requirements, including those for file naming.

**Answer**

Thank you for pointing this out. We had ensured that the manuscript met PLOS ONE style requirements, including file naming.

**Point 2**

As per the journal’s editorial policy, please include in the Methods section of your paper:

1) your reasons for your delay in registering this study (after enrolment of participants started).

2) confirmation that all related trials are registered by stating: “The authors confirm that all ongoing and related trials for this drug/intervention are registered”.

**Answer**

Thank you for pointing this out. We have added a note about the reasons for our delay in registering for this study (after enrolment of participants started) and confirmation that all related trials are registered.

**Materials and Methods (Ethics Approval)**

**Line 213:**

The study protocol was approved by the Ethics Committee of Kanazawa University Hospital (Approval No.: 2021-222-113867) and was conducted in accordance with the Declaration of Helsinki. […] **This pilot study was conducted as a preliminary investigation while creating a clinical trial protocol for patients with CKD**. Although ethical approval had been obtained **from the ethics committee** prior to the pilot study, **trial registration was not considered mandatory**. Since the UMIN-CTR accepts registrations after the clinical trial has ended, the trial was registered after its completion. **The authors confirm that all ongoing and related trials for this intervention are registered.** Such late registration does not affect the validity of study results or the safety and rights of participants.

**Point 3**

Funder's roles: Funding and advice for the overall research. Please state what role the funders took in the study. If the funders had no role, please state: "The funders had no role in study design, data collection and analysis, decision to publish, or preparation of the manuscript." If this statement is not correct you must amend it as needed. Please include this amended Role of Funder statement in your cover letter; we will change the online submission form on your behalf.

**Answer**

Thank you for pointing this out. We have added a note about the funder's roles to the cover letter.

**Cover letter**

We have approved the manuscript and agree with submission to PLOS ONE. There are no conflicts of interest to declare. **This study was conducted with a grant from Japan Agency for Medical Research and Development (AMED). The funders had no role in study design, data collection and analysis, decision to publish, or preparation of the manuscript.**

# **Author's reply to the review report (reviewer 1)**

## **Major Points for Improvement**

**Point 1**

The lack of a control group makes it difficult to attribute observed outcomes solely to sake lees intake. Including a placebo or “no intervention” group would strengthen causal inferences.

**Answer**

Thank you for your comment. We think the points you raise are very important.

Because there are no previous studies that have investigated changes in fecal uremic toxins in CKD patients due to the sake lees intake, it was necessary to evaluate whether the sake lees intake would change the fecal uremic toxins of non-CKD patients, including safety. Since this was an exploratory study, we considered a single-arm study to be appropriate from ethical considerations and conducted the study without setting up a control group. We have added this point to the “Materials and Methods” and “Limitations” section.

**Materials and Methods (Study Design and Participants)**

**Line 73:**

This pilot study investigated the effects of sake lees intake on fecal uremic toxin levels in individuals with chronic constipation. It employed an open-label, single-arm, before and after design without a control group. **No previous studies have investigated changes in fecal uremic toxins in patients with CKD after sake lees intake. Therefore, evaluating whether sake lees intake changes fecal uremic toxins in non-CKD patients is necessary. Because this was an exploratory study, a single-arm study design was considerably appropriate based on ethical considerations, and the study was conducted without a control group [13]**. A sample size estimation was not performed due to the absence of prior data on effect size.

[13] Wang M, Ma H, Shi Y, et al. Single-arm clinical trials: design, ethics, principles. BMJ Support Palliat Care. 2024;15: 46-54.

**Discussion**

**Line 364:**

This study has several limitations. First, **this was a pilot study that used a single-arm design without a control group, potentially limiting the interpretation. This study was conducted as an early phase of clinical trial implementation for patients with CKD. The primary objectives were to evaluate the feasibility, collect sample size information, and assess the potential effects of sake lees intake on the selected biomarkers. In this early exploratory phase, a single-arm design was used for feasibility and ethical considerations.** Second, […]

**Point 2**

Because participants were aware of their treatment status, subjective measures (e.g., constipation severity scores) may be influenced by expectancy bias. Blinded designs or at least blinded endpoint assessments would help mitigate this risk.

**Answer**

Thank you for your comment. We agree that blinding is important for subjective measures. We have revised the “Limitations” section that the results of the subjective measures, the CSS and the PAC-QOL scores, may be influenced by expectancy bias.

**Discussion**

**Line 371:**

This study has several limitations. [...] Second, as an open-label study, the CSS and PAC-QOL scores, which were evaluated by participants using subjective measures, may have been influenced by **expectancy bias (a type of information bias)**.

**Point 3**

The manuscript acknowledges that the sample size was not determined based on effect size or power calculations. Providing a more robust sample size calculation—based on preliminary data or existing literature—will enhance the credibility of the results.

**Answer**

Thank you for your comment. We have added a “Discussion” section with sample size calculations based on existing literature.

**Discussion**

**Line 371:**

This study has several limitations. […] Third, **we could not determine the sample size based on effect size or statistical power before the study owing to feasibility constraints. The small sample size may have reduced the statistical power and increased the likelihood of distortion due to outliers or random fluctuations. Furthermore, the participants do not accurately reflect the population, potentially limiting the generalizability of the findings. Post hoc power analysis revealed that, assuming a difference of 50 µg/g in fecal indole (approximately half of the 112 µg/g difference reported in a previous randomized controlled trial [30]) and extrapolating the standard deviation (30 µg/g), our actual sample size of the clinical trials in patients with CKD (n = 11 and 13) would yield a statistical power of 97%.**

[30] Park YE, Kim MS, Shim KW, et al. Effects of Lactobacillus plantarum Q180 on Postprandial Lipid Levels and Intestinal Environment: A Double-Blind, Randomized, Placebo-Controlled, Parallel Trial. Nutrients. 2020;12: 255.

**Point 4**

While fecal uremic toxin changes are an interesting surrogate, the manuscript would benefit from clarifying how well they correlate with blood levels or clinical outcomes. It would be worthwhile to measure blood uremic toxin levels or additional relevant biomarkers to strengthen the link between gut environment changes and systemic impacts.

**Answer**

Thank you for your comment. We think it is important to observe changes in uremic toxins in the blood and feces. It is known that fecal indole and blood indoxyl sulfate are correlated in CKD patients, and lowering fecal indole is one of the treatment strategies. However, the participants of this study were non-CKD. We suspect that uremic toxins such as indoxyl sulfate in the blood are excreted by the kidneys and the values do not change. We have added this information to the “Materials and “Methods” section.

**Materials and Methods (Primary and Secondary Endpoints)**

**Line 129:**

The primary endpoints were the percentage changes in fecal indole and p-cresol levels from baseline. [...], and adherence to sake lees intake were assessed. **Regarding blood uremic toxins, fecal and blood uremic toxins correlate in patients with CKD, and lowering fecal uremic toxins is a treatment strategy [1]. However, the participants were non-CKD patients, and the levels of blood uremic toxins, such as indoxyl sulfate, would not change, potentially because of excretion from the kidneys. Therefore, the blood uremic toxin levels were not measured.**

[1] Rysz J, Franczyk B, Lawinski J, Olszewski R, Cialkowska-Rysz A, Gluba-Brzozka A. The Impact of CKD on Uremic Toxins and Gut Microbiota. Toxins (Basel). 2021;13: 252.

**Point 5**

Factors such as fluid intake, physical activity, and timing of sake lees consumption could significantly influence constipation and gut microbiome outcomes. These should be explicitly addressed in methods and discussion sections to rule out confounding effects.

**Answer**

Thank you for your comment. We have added information on fluid intake, physical activity and timing of sake lees consumption to the “Materials and Methods” and “Discussion” section.

**Materials and Methods (Dietary Treatment)**

**Line 107:**

The daily sake lees intake amounts were set at 25 g and 50 g, based on a previous study. […] Since fecal indole and p-cresol are metabolites produced by gut bacteria from dietary protein, participants were instructed to maintain their usual eating habits throughout the run-in and treatment periods. **Additionally, they were instructed to maintain their usual fluid intake and physical activity habits considering these can influence constipation.**

**Discussion**

**Line 342:**

Constipation improved after consuming sake lees in this study. Previous studies have found that sake lees consumption increased defecation frequency and improved stool characteristics. […] The lack of differences in the CSS and PAC-QOL scores based on sake lees amount is unclear, but it may be due to the short duration of the dietary treatment in this study. **Because this was an open-label, single-arm study, changes in the participants’ usual habits, including diet, fluid intake, and physical activity, which can influence constipation, might have occurred because of information bias. Additionally, because the timing of sake lees intake was not fixed to ease implementation, this might have influenced the results.**

## **Minor Points for Improvement**

**Point 1**

Proofread for minor typos (e.g., “To data” in line 61 should be “To date”), consistent terminology (e.g., “sake lees” vs. “Sake lees”), and uniform tense throughout the manuscript.

**Answer**

Thank you for your comment. We corrected any typos or consistent terminology and revised the tense of the entire manuscript. The word “sake lees” is treated as a common noun in this paper, and we have standardized the notation as “Sake lees” at the beginning of a sentence and “sake lees” in the middle of a sentence.

**Point 2**

Ensure that all figures and tables are clearly labeled with descriptive titles and legends, and that axis labels are self-explanatory. Double-check that the numbering of figures and references in the main text aligns with the final layout.

**Answer**

Thank you for your comment. We have rechecked the titles, legends and axis labels for the figures and tables. We have also rechecked whether the figures and tables in the main text correspond to the reference numbers.

**Point 3**

Verify that references follow the journal’s style guide and that all in-text citations match the reference list. In particular, older references might be updated with more recent studies addressing gut microbiota, chronic constipation, or CKD-related dietary interventions.

**Answer**

Thank you for your comment. We have updated references and verified that all in-text citations match the reference list.

**Point 4**

Although the authors mention participants could consume sake lees in various ways, it may help to standardize or at least detail these methods to reduce variability and better interpret outcomes.

**Answer**

Thank you for your comment. We think the points you raise are important. We have added a section on “Discussion” regarding the standardization of the way and timing of sake lees intake.

**Discussion**

**Line 357:**

Long-term commitment is required for dietary treatments, so clinical studies must assess adherence and potential side effects. In this study, both the 25-g and 50-g sake lees intake groups showed good adherence. **Participants consumed sake lees based on their preference. Standardizing consumption methods and timing may improve outcome interpretation.** No adverse events such as diarrhea or constipation were reported by participants. Previous studies have also found no adverse events linked to sake lees intake. In contrast, current drug treatments targeting intestinal uremic toxins in CKD patients often face low compliance. If sake lees dietary treatment proves effective for CKD patients, it could complement these drug treatments.

# **Author's reply to the review report (reviewer 2)**

**Point 1**

This study lacks a control group. The absence of a control group limits the ability to compare the effects of sake lees against a placebo, reducing the robustness of causal inferences.

**Answer**

Thank you for your comment. We think the points you raise are very important. However, this pilot study was conducted on an urgent basis during the process of creating a clinical trial protocol for CKD patients, and it was not possible to set up a control group from the perspective of the feasibility of the study. We have added this point to the “Limitations” section.

**Discussion**

**Line 364:**

This study has several limitations. First, **this was a pilot study that used a single-arm design without a control group, potentially limiting the interpretation. This study was conducted as an early phase of clinical trial implementation for patients with CKD. The primary objectives were to evaluate the feasibility, collect sample size information, and assess the potential effects of sake lees intake on the selected biomarkers. In this early exploratory phase, a single-arm design was used for feasibility and ethical considerations.** Second, […]

**Point 2**

The study included only eight participants, which limits the statistical power and generalizability of the findings. Small sample sizes also increase the risk of outliers disproportionately influencing results.

**Answer**

Thank you for your comment. We have added to the “Limitations” that due to the small sample size of the study, the results may be susceptible to outliers, limiting the generalizability of the findings.

**Discussion**

**Line 373:**

This study has several limitations. […] Third, we could not determine the sample size based on effect size or statistical power before the study owing to feasibility constraints. **The small sample size may have reduced the statistical power and increased the likelihood of distortion due to outliers or random fluctuations. Furthermore, the participants do not accurately reflect the population, potentially limiting the generalizability of the findings.**

**Point 3**

The study duration is short. The six-week intervention may not have been long enough to observe sustained changes in gut microbiota composition or lasting health benefits.

**Answer**

Thank you for your comment. As you pointed out, six-week intervention may be short to observe changes. Based on the results of this pilot study and previous studies, the longer (8 weeks) clinical trial for CKD that was conducted after this study. We have added to the “Limitations” that the six-week intervention may not have been long enough to observe sustained changes in the gut microbiome or lasting health benefits.

**Discussion**

**Line 352:**

In this study, sake lees intake changed the phylum proportions of the fecal microbiome, which aligns with previous research indicating that fermented foods, such as sake lees, impact the gut microbiome [26, 27]. For instance, an increase in the Firmicutes phylum, which is considered beneficial for health, was observed. One possible explanation for this change could be the dietary fiber content in sake lees [28]. Sake lees is a fermented food that contains a variety of bacteria and may contribute to changes in the fecal microbiome [26, 27]. **Although this study was a 6-week intervention, our findings indicate the possibility that longer-term interventions can sustain changes in the gut microbiome and provide lasting health benefits.**

**Point 4**

The reliance on self-reported metrics, such as the Constipation Scoring System (CSS) and quality-of-life questionnaires, introduces the potential for bias and variability in participant responses.

**Answer**

Thank you for your comment. We have added to the “Limitations” section that the results of the subjective measures, the CSS and the PAC-QOL scores, may be influenced by expectancy bias.

**Discussion**

**Line 371:**

This study has several limitations. […] Second, as an open-label study, the CSS and PAC-QOL scores, which were evaluated by participants using subjective measures, may have been influenced by **expectancy bias (a type of information bias).**

**Point 5**

Variables such as diet, physical activity, hydration levels, and other lifestyle factors that could influence constipation and gut microbiota were not controlled or systematically analyzed.

**Answer**

Thank you for your comment. We have added information on diet, physical activity, fluid intake and other lifestyle factors to the “Discussion” section.

**Discussion**

**Line 342:**

Constipation improved after consuming sake lees in this study. Previous studies have found that sake lees consumption increased defecation frequency and improved stool characteristics. […] The lack of differences in the CSS and PAC-QOL scores based on sake lees amount is unclear, but it may be due to the short duration of the dietary treatment in this study. **Because this was an open-label, single-arm study, changes in the participants’ usual habits, including diet, fluid intake, and physical activity, which can influence constipation, might have occurred because of information bias. Additionally, because the timing of sake lees intake was not fixed to ease implementation, this might have influenced the results.**
