# Supplementary material for: Effects of sake lees intake on fecal uremic toxins, plasma D-alanine, constipation, and gut microbiome in healthy adults: A single-arm clinical trial
Source: PLoS One. 2025 Jun 3;20(6):e0325482. doi: 10.1371/journal.pone.0325482 (PMC12133169; doi:10.1371/journal.pone.0325482)
Supplement: Attachment — Submitted filename: Response_to_the_Reviewer_auresp_2.docx [file pone.0325482.s012.docx]

# **Author's reply to the review report (reviewer 3)**

**Comment 1**

The authors employed a single-arm trial without a control group. Even though some researchers feel it is not mandatory, a comparison or control group is often beneficial in pilot studies to help assess the feasibility, effectiveness, and robustness of the study design and protocols before a larger, full-scale study. In addition, a control group allows researchers to assess whether the proposed intervention or method is likely to be effective and practical. It is possible to observe a change in a single-arm trial, but it becomes extremely difficult to gauge the likely impact of the proposed intervention (sake lees intake) without a control group during the pilot study in a full-scale study. The implication of doing a single arm without a control group must be thoroughly discussed.

**Answer**

Thank you for your comment. This study was conducted with the aim of exploratorily observing changes in uremic toxins due to sake lees intake, which has not been previously studied or reported, and to assess the feasibility of conducting such study. We consider that the potential benefits of sake lees intake should be verified in future studies using a control group. As the reviewer pointed out, the implications of conducting a single-arm study without a control group must be thoroughly discussed. Based on this comment, we have added it to the “Discussion” section.

**Discussion**

**Line 315**

This study was a single-arm, before and after pilot study involving eight individuals with chronic constipation, serving as a preliminary step for a clinical trial in patients with CKD. After consuming 25 g or 50 g of sake lees daily for 6 weeks, changes in fecal uremic toxin levels were observed. These consumption patterns also influenced plasma D-amino acid levels, constipation status, and the fecal microbiome. **Although these findings indicate the therapeutic effects of sake lees, further studies with control group and sufficient sample size are needed for confirmation.**

**Comment 2**

Since there was no control group, the conclusion drawn from the pilot study must be interpreted with caution, and this must be clearly stated.

**Answer**

Thank you for your comment. Based on your suggestion, we have clearly stated in the conclusion that the results of this pilot study must be interpreted with caution due to the lack of a control group.

**Conclusions**

**Line 397**

Sake lees intake may reduce fecal uremic toxins, increase plasma D-alanine levels, improve constipation, and alter the gut microbiome. **Although these results are considered meaningful, caution is needed in interpreting the therapeutic effects. Therefore**, further studies **with a control group** are needed to assess the impact of sake lees on uremic toxins and the gut microbiome in patients with CKD.

**Comment 3**

The authors indicated that it was an exploratory study, which was why no control group was studied, but added that the study will lay the groundwork for a clinical trial targeting chronic kidney disease (CKD) patients. This is the more reason why control patients should have been included: if the intervention (sake lee intake) is potentially harmful or has unknown risks, a control group can help ensure that participants in the main study are not exposed to unnecessary risks.

**Answer**

Thank you for your comment. This study was conducted exploratory to obtain basic information for clinical trials targeting CKD patients. We recognize your concern that a control group would be important, especially to assess potential risks or harms of the intervention (sake lees intake). However, sake lees has been consumed in Japan for centuries, and its safety has been confirmed in previous studies involving healthy individuals. For this reason, we did not conduct a safety evaluation with a control group in this study. We have added a statement to the manuscript noting that we plan to include a control group for safety evaluation in the upcoming clinical trial involving CKD patients.

**Discussion**

**Line 384**

This study has several limitations. First, […] Fifth, factors influencing constipation improvements, such as fluid intake and physical activity, were not considered in this study.

**Sixth, safety evaluation could not be conducted because of the absence of a control group. Sake lees have been consumed in Japan for centuries, and its safety has been confirmed in trials involving healthy individuals [8]. In the next clinical trial targeting patients with CKD, a control group must be included to assess safety.** Lastly, changes in fecal uremic toxins were used as a surrogate for blood uremic toxins, which may not fully reflect changes in blood levels.

**Comment 4**

Authors stated that sample size was not performed due to the absence of prior data on effect size. However, the authors could have adopted the approach of Cohen’s et al (Cohen classified effect sizes as small (d = 0.2), medium (d = 0.5), and large (d ≥ 0.8) to simulate different sample sizes taking into account the budget.

Cohen, J. (2016). Things I have learned (so far). American Psychological Association.

Not estimating sample size with the justification that there was no effect size from previous studies is problematic.

**Answer**

Thank you for your comment. We did attempt to adopt Cohen's approach, but using the assumed effect sizes (d = 0.2, 0.5, 0.8) did not result in a feasible sample size given the constraints of research resources (budget and period). We have added a note to this effect in the “Materials and Methods” section.

**Materials and Methods**

**Study Design and Participants**

**Line 78**

This pilot study investigated the effects of sake lees intake on fecal uremic toxin levels in individuals with chronic constipation. […] Because this was an exploratory study, a single-arm study design was considerably appropriate based on ethical considerations, and the study was conducted without a control group [13]. A sample size estimation was not performed due to the absence of prior data on effect size. **An attempt was made to determine the sample size using hypothetical effect size; however, the calculated sample size was not feasible for research concerning budget and time period.**

**Comment 5**

There was no rigorous power analysis, but the authors used a one-sample t-test to test whether the mean value of the primary outcome measure equals zero. How did the authors determine whether the study was powered enough to test that hypothesis? A similar comment applies to the use of repeated-measure ANOVA.

**Answer**

Thank you for your comment. Based on your suggestion, we conducted a post hoc power analysis for the sample size of this study (n = 8). The results indicated that small to moderate effects (d = 0.2–0.5) could not be adequately detected, and even large effects (d = 0.8) were a power of about 48%. However, this study was exploratory in nature and aimed to observe changes in fecal uremic toxins due to sake lees intake, requiring careful interpretation of the therapeutic effects. We have added it to the “Discussion” section.

**Discussion**

**Line 315**

This study was a single-arm, before and after pilot study involving eight individuals with chronic constipation, serving as a preliminary step for a clinical trial in patients with CKD. After consuming 25 g or 50 g of sake lees daily for 6 weeks, changes in fecal uremic toxin levels were observed. These consumption patterns also influenced plasma D-amino acid levels, constipation status, and the fecal microbiome. **Although these findings indicate the therapeutic effects of sake lees, further studies with control group and sufficient sample size are needed for confirmation.**
